# Radiological Cardiothoracic Ratio in Evidence-Based Medicine

**DOI:** 10.3390/jcm10092016

**Published:** 2021-05-08

**Authors:** Krystian Truszkiewicz, Rafał Poręba, Paweł Gać

**Affiliations:** 1 Center for Diagnostic Imaging, University Clinical Hospital in Wrocław, Borowska 213, PL 50-556 Wroclaw, Poland; ktruszkiewicz@usk.wroc.pl; 2 Department of Internal Medicine, Occupational Diseases and Hypertension, Wroclaw Medical University, Borowska 213, PL 50-556 Wroclaw, Poland; rafal.poreba@umed.wroc.pl; 3 Centre for Diagnostic Imaging, 4th Military Hospital, Weigla 5, PL 50-981 Wroclaw, Poland; 4 Department of Hygiene, Wroclaw Medical University, Mikulicza-Radeckiego 7, PL 50-368 Wroclaw, Poland

**Keywords:** cardiothoracic ratio, chest radiography, heart enlargement

## Abstract

The cardiothoracic ratio (CTR), expressing the relationship between the size of the heart and the transverse dimension of the chest measured on a chest PA radiograph, is a commonly used parameter in the assessment of cardiomegaly with a cut-off value of 0.5. A value of >0.5 should be interpreted as enlargement of the heart. The following review describes the current state of available knowledge in terms of contentious issues, limitations and useful aspects regarding the CTR. The review was carried out on the basis of an analysis of scientific articles available in the PubMed database, searched for using the following keywords: “CTR”, “cardiothoracic ratio”, “cardiopulmonary ratio”, “cardiopulmonary index”, and “heart-lung ratio”. According to the accumulated knowledge, the CTR can still be used as an important parameter that can be easily determined in establishing enlargement of the heart. However, an increased CTR does not directly relate to heart function. In the era following the development of diagnostic methods such as computed tomography, magnetic resonance imaging, and ultrasonography, CTR modifications based on these methods are used with varying clinical usefulness. It is important to consider the definition of the CTR and remember to base measurements on PA radiographs, as attempts to mark it in other projections face many limitations.

## 1. Introduction

**Figure 1:**
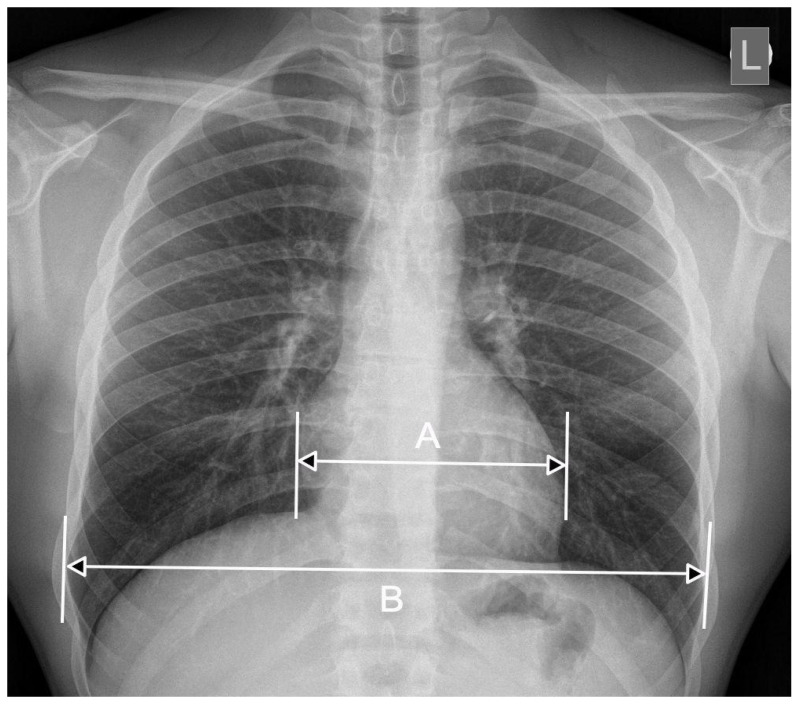
The method of determining the CTR. The CTR is determined on the basis of the ratio of the transverse heart dimension [A] to the transverse dimension of the chest (internal ribs) [B] measured on the radiograph in the chest PA projection: CTR = A/B.

A chest PA examination, apart from its usefulness in oncology, pulmonology, pediatrics, etc., is also used as a screening tool to assess the size of the heart′s silhouette. A commonly used parameter for the heart′s evaluation is the cardiothoracic ratio (CTR), first described in 1919 [1]. The cardiothoracic ratio is defined as the ratio of the greatest transverse dimension of the heart to the greatest transverse dimension of the chest cavity measured to the inner surface of the ribs on the PA radiograph [1] (Figure 1). This parameter is easy to determine and does not significantly extend the interpretation of X-ray images, especially with the capabilities of modern DICOM image viewer tools, which may be one of the reasons for not adopting the automatic CTR measurement method [2]. Normal values range between 0.42 and 0.50, which should not be presented as a percentage, but as a ratio. A value above 0.50 is considered abnormal and may indicate cardiomegaly [3]. Unfortunately, when assessing the cardiac silhouette on a chest radiograph, it can be difficult to distinguish true enlargement of the heart from enlargement as a result of pericardial disease (e.g., effusion). Despite this, the universality of the CTR in various populations can be proved, for example, a study conducted in Ghana on a group of almost 2000 patients demonstrated that the mean (normal) CTR in the population was <0.5. According to the investigators, the mean CTR for their study population was 0.452. The researchers also found a difference in the mean CTR value for women (0.467) and men (0.459) and established its variability with age (the index increases with age). Patients also showed an increase in chest width until the sixth decade of life, after which this index decreased [4].

The assessment of the heart’s silhouette on a chest radiograph, in addition to the CTR mentioned above, may allow us to obtain additional, clinically useful information at a much lower dose of ionizing radiation (between 80 and 100 times) compared with computed tomography. Assessing the silhouette of the heart enables, among others suspicion of aortic dilatation, information about the presence of rings of implanted valves, calcifications in the projection of the valve fields, assessment of the position of pacemaker electrodes, etc. Additionally, it should be remembered that chest radiographs are not performed only to determine the CTR; it is determined along with other diagnostic purposes of the study.

## 2. CTR—The Importance of Radiograph Projection

The cardiothoracic ratio should only be determined by a PA radiograph projection. Although in the literature we find appropriate values of the coefficient, for example, for the AP projection [5], we should approach them with care and be aware of their limitations [5]. Chest radiograph, and thus also CTR determination, is an easy and beneficial parameters in patient assessment in hospital emergency departments (HEDs) and in the course of treatment and patient care in intensive care units (ICUs) [6]. The clinical condition of such patients, however, often does not allow for the execution of a chest radiograph in the PA projection, which is most often substituted with bedside examinations of the AP projection. We must remember the basic limitations related to determining the CTR in such clinical situations. First, the width of the heart, located in the mediastinum closer to the anterior chest wall, is greater if the X-ray beam is directed from the front to the rear. Secondly, the distance between the radiation source (lamp) and the detector (cassette) is shorter than when taking PA radiographs (for PA radiographs, approx. 180–200 cm) [7], which results in the obtained radiograph enlarging both the silhouette of the heart and the transverse dimensions of the chest. Thirdly, the clinical condition of such patients often does not allow them to perform a full inhalation or to maintain it for an appropriate time, which results in unreliable measurements of the transverse dimensions of the chest [8]. It should also be remembered that the conditions for producing bedside radiographs are highly variable.

The above-described variables and the inconvenience in determining the CTR via AP radiographs were reflected in numerous research studies that sought methods to determine this easy parameter. Chon et al. [8] proposed the corrected CTR, calculated from the formula they proposed. However, this measurement requires access to a patient′s prior PA radiograph, and the sensitivity and specificity of this measurement were determined to be 61% and 54%, respectively. The study was also limited by the small validation group (18 patients with echocardiographic features of heart failure and 17 without such features).

Kabala and Wilde [9] proposed, based on the results of their work (and on a tomographic and radiographic model), to define the enlargement of the heart silhouette based on AP radiographs as CTR > 55% and the width of the heart shape >165 mm for men and 150 mm for women (sensitivity 92%, specificity 96%). A limitation of this study was the selection of the group. The researchers selected people with histories suggesting symptoms of heart failure, including their radiological features, and the results were not correlated with other diagnostic methods, e.g., echocardiography.

## 3. CTR—The Meaning of the Breathing Phase, the Anterior-Posterior Heart Dimension

Another variable that significantly affects the CTR value is the respiratory phase; the silhouette of the heart is larger during exhalation and smaller if the radiograph of the same patient is taken during inspiration. The dimensions of the chest are also variable in both breathing phases, but the work of Tomita et al. [10], based on the multi-parameter study of the dimensions of the chest and heart silhouette, the computed tomography of the chest in inspiration and exhalation, and the determination of the CTR in both phases, proved that the developed CTR under these conditions is significantly higher (*p* < 0.0001) in exhalation than in inspiration. This indirectly proves a significant disproportion of changes in lung size and the cardiac silhouette during respiratory phases, and emphasizes the importance of the pre-evaluation of data prior to CTR determination. These data are also confirmed in populations of children. The textbook Pediatric Radiology [11] describes significant changes in the size of the heart silhouette depending on the respiratory phase, using the example of computed tomography of the chest performed on a crying child. As in the adult population, the CTR was higher during exhalation and significantly lower during inspiration. Due to the inability to hold breath in the population of newborns, infants, and young children, the CTR is of limited value in the diagnosis and evaluation of congenital heart defects associated with enlargement. The situation is quite different for the adult population with congenital heart disease undergoing repair surgery. In a group of over 3000 adults with congenital heart disease, the CTR was shown to be a simple, reproducible factor correlating with cardiac function and may be an independent predictor of long-term mortality [12].

**Figure 2:**
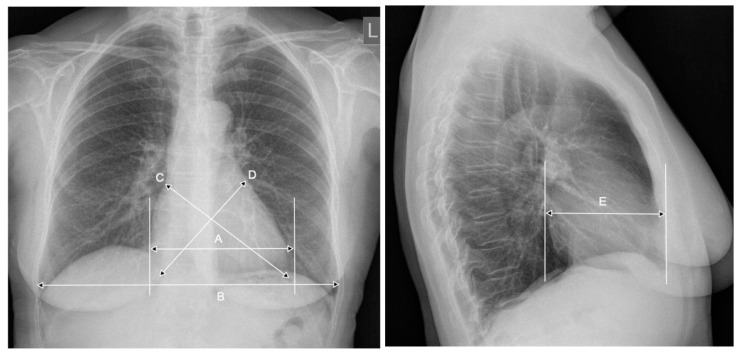
Additional parameters taken into account in determining the heart volumes in the Whitehall Study: A—the transverse dimension of the heart, B—the transverse dimension of the chest, C—the long axis of the heart, D—the broad axis of the heart, and E—the horizontal axis of the heart.

The CTR is determined (according to its definition) on the basis of a flat chest PA radiograph. Assessment of cardiomegaly on the basis of the CTR may not always be appropriate due to the inability to account for changes in the anterior-posterior heart size and changes in the long axis of the heart. Researchers from Japan [13] showed that the CTRs in these patients is influenced by the transverse rotation of the heart in the counterclockwise dimension. Such rotation cannot be reliably assessed on a flat radiograph, and the study did not find an appropriate method of including it in the CTR assessment literature. In the 25 year prospective Whitehall Study, researchers added heart measurements in three axes to the classic measurement of the CTR: the long axis, the broad axis determined on the PA radiograph, and the horizontal axis determined in the anterior-posterior dimension on the radiograph in the profile projection. Using the obtained values in an appropriate mathematical algorithm, they determined the heart volume and correlated it with the body surface of the relative heart volume [14], (Figure 2). The study concluded that there was no higher value of the relative heart volume and thus determined the classical CTR for predicting mortality from ischemic heart disease [14].

## 4. CTR as a Prognostic Factor

The CTR was found to be one of the prognostic factors in several groups of diseases. An example is the population of people undergoing hemodialysis. A large, prospective, cohort study on a group of almost 3500 people with a four-year follow-up showed that in people undergoing hemodialysis, a higher CTR is associated with a higher risk for both general and cardiovascular reasons [15].

In an animal model, it was shown that chronic vitamin D deficiency is associated with myocardial remodeling, leading to fibrosis, myocardial hypertrophy, myocarditis, and heart systolic disorders. Based on these observations, a study was conducted on a group of chronic hemodialysis patients to assess the impact of vitamin D deficiency in conjunction with CTR determination on the prognosis in this group. The study found that the CTR was higher in patients with vitamin D deficiency, and may be an independent prognostic factor of vitamin D deficiency in this group of patients [16].

It was also shown in the group of hemodialysis patients that a CTR > 55% is one of the most important independent factors influencing the two-year all-cause mortality rate [17]. Hence, the suggestion to analyze the heart condition in patients at the start of hemodialysis. In a large group of almost 1800 people with a four-year follow-up, the CTR as a simple factor was shown to be a good prognostic factor for poor prognosis in patients with rheumatic heart disease undergoing heart valve replacement surgery. A baseline CTR > 0.6 was shown to be an independent, poor prognostic factor for death during perioperative hospitalization and during the first year following valve replacement [18].

## 5. CTR and Heart Function

At the time of the development of the CTR, and for many years of its use, values > 0.5 were interpreted not only as enlargement of the heart but also as impaired heart function (left and/or right ventricle). Interesting conclusions were drawn by researchers from London, who related the CTR measured on radiographs of PA and AP to the measurement data obtained in echocardiography in patients treated in the emergency mode. The first conclusions were similar to those described several times above and concern a limited CTR value determined on AP radiographs; the researchers demonstrated that this a CTR was not correlated with left or right ventricular dysfunction. According to the researchers, the CTR determined on the PA radiograph has moderate sensitivity and specificity but low positive predictive value in the diagnosis of right or left ventricular systolic disorders. The researchers highlighted the problem of a lack of correlation between a “big heart” and its dysfunction. This is probably due to the development of diagnostics and the implementation of prior treatment; therefore, a large heart cannot be equated to a heart with the presence of dysfunction [19]. It is important to know whether the CTR may normalize or decrease with the introduction of treatment and whether the CTR may be a prognostic factor in this situation. Breur et al. assessed the effect of pacemaker therapy in patients with isolated congenital total atrioventricular block. The following were taken as heart size markers: the left ventricular end-diastolic diameter (LVEDD), the left ventricular shortening fraction (FS), and the CTR. The study showed that in patients without pacemaker implantation, CTR increases by 0.02%/month, and in patients with pacemakers, it decreases by 0.19%/month; the difference was statistically significant. It was proven that early pacemaker implantation results in a reduction in the size of the heart, also in the assessment of the CTR. The conclusion was that the progressive increase or persistently high CTR (and LVEDD) values in asymptomatic patients should be taken into account when implanting an electrostimulator in order to avoid the development of congestive heart failure [20].

**Figure 3:**
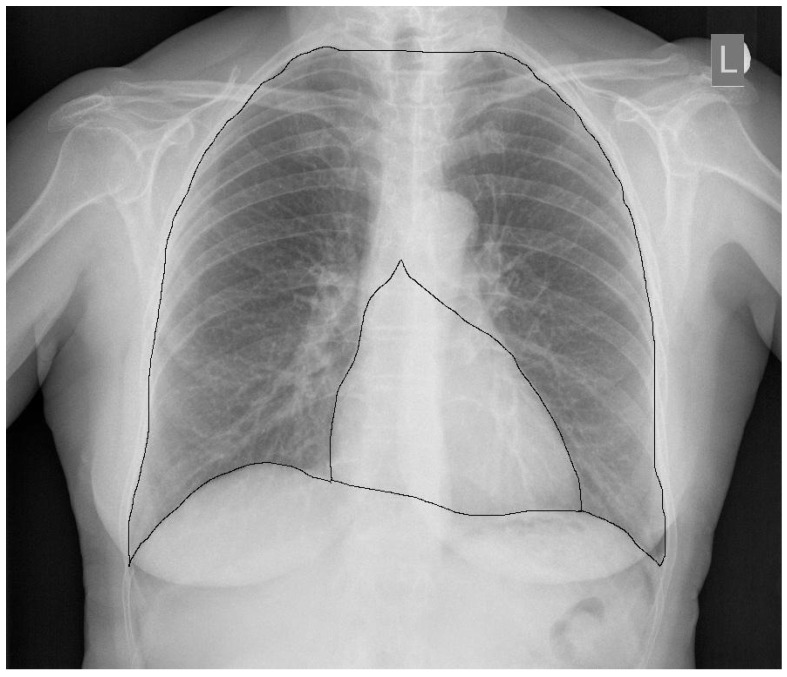
Demarcation lines delineating the surface areas used to determine the 2D CTR.

Researchers from Ireland tried to link the classical one-dimensional CTR and their two-dimensional CTR with the left ventricular ejection fraction [21]. The 2D CTR was defined as the ratio of the number of pixels covering a previously determined area of the heart (the cardiac area) to the number of pixels covering the surface of the lung fields and the middle shadow (the whole thorax area) (Figure 3). The ejection fraction was determined by radioisotope studies. The researchers demonstrated a strong correlation between the classic CTR and 2D CTR (*r* = 0.82); however, on comparing both coefficients with the left ventricular ejection fraction, they concluded that an increased 2D CTR better correlated with a reduced ejection fraction (*r* = −0.52) than the classic CTR (*r* = −0.45). They proposed a value of a 0.23 2D CTR (or a ratio of 1/4) or less as the correct value, which should indirectly indicate a fraction of ≥55%.

On the basis of computed tomography studies, it was shown that the size of the left ventricle can be more accurately predicted on the basis of a simple and highly reproducible measurement of the left ventricular surface on axial chest computed tomography scans than on the CTR [22]. Images for measurements of the left ventricular surface were obtained in two groups: gated ECGs of CT examinations with contrast enhancement and gated ECGs of examinations without the use of a contrast agent; CTR measurement was performed on topographic scans. Despite the good and repeatable results of measurements of the left ventricular parameters thus obtained, the method of its determination should be considered a limitation when comparing them with the CTR (the limitations of the CTR determined in this way are described later in this study).

However, it was shown that a baseline CTR > 0.5 in patients with chronic heart failure is associated with higher mortality and higher hospitalization rates, and a routine PA radiograph along with CTR measurement was indicated as an important part of the management of such patients. The CTR in this case can be used as a value to stratify cardiovascular risk [23].

## 6. CTR in the Pediatric Population

Due to the above-described limitations of the use of the CTR in children, there are several studies investigating this problem in the literature.

Canadian researchers from Toronto conducted a retrospective study of 127 children (mean age 11.2 ± 5.5 years) with the most common structural and myopathic congenital myocardial defects, in which they checked the correlation of the CTR with the volume of the heart cavities measured by magnetic resonance [24]. It was shown that a higher CTR is associated with an increased total heart volume in children with aortic and pulmonary valve insufficiency; however, significant differences in the volume of individual chambers and the size of the CTR limit its use in monitoring the patient′s heart condition. The CTR did not correlate with ventricular volumes in patients with left-right shunt and hypertrophic cardiomyopathy (HCM). In recent cases, the additional coefficient determined on the lateral radiograph did not offer additional value in assessing the size of the heart.

In an interesting case report of an eight-year follow-up on a girl with isolated congenital complete atrioventricular block (ICCAVB) with long-term serum NT-proBNP measurement in conjunction with CTR measurement [25], the girl was born by caesarean section in the 36th week of pregnancy due to abnormal HR values, and then she was diagnosed with ICCAVB, without the need for pacemaker implantation. NT-proBNP is one of the end products (with biologically active BNP) of pre-proBNP metabolism, released by the muscle cells of the heart ventricles when their stress increases (overload or volume). It became natural to associate it with the CTR, which is also used to assess patients with heart failure. In the described case, a large positive correlation was found between the CTR and the level of NT-proBNP in the serum (*p* < 0.05). As a conclusion, it was proposed that in children with ICCAVB, measuring the CTR during their follow-up cab be a useful tool in assessing the load on the heart.

The CTR measurement was found to have a role during the fetal stage. Obstetricians from Thailand performed CTR measurements using ultrasound in fetuses from pregnancies at risk of Bart′s hemoglobin disease and demonstrated that such measurements have a great prognostic value [26]. Researchers demonstrated a significant increase in the CTR in affected fetuses during the late first/early second trimester of pregnancy. They showed the 13th week of pregnancy as the median increase in the CTR, and all fetuses with the disease were selected up to 23 weeks of gestation. The study also assessed the flow parameters of the middle cerebral artery (MCA-PSV) and showed that only slightly more than 9% of the fetuses developed abnormal flow in this artery before the CTR increased. The conclusion drawn was that the discovery will help identify Bart’s hemoglobin disease in earlier pregnancy and, therefore, more appropriate individualized pregnancy ultrasound regimens can be developed.

Another example of the use of a modified form of CTR in fetal life is the prediction of lethal obstruction of the pulmonary veins in fetuses diagnosed by prenatal ultrasound of a single ventricle with the coexistence of total abnormal pulmonary vein connection (TAPVC). Researchers from Japan using the cardiothoracic area ratio (CTAR), calculated as the ratio of the heart area to the thoracic area on the ultrasound data, showed that a reduced prenatal CTAR is a good predictor of lethal obstruction of the pulmonary veins after birth [27]. Such information allows time for the preparation of parents and, above all, for the development of an appropriate procedure to follow after the birth of a child with this syndrome of defects.

## 7. Post-Mortem Examinations—An Attempt to Evaluate CTR

The CTR is also applicable in autopsy. A benefit of such studies is the negligible harmful effect of ionizing radiation on the study group. Researchers from Switzerland, based on post-mortem computed tomography studies, proposed the new CTR formula for the diagnosis of cardiomegaly [28]. In determining the new CTR formula, apart from heart and chest measurements in axial scans of computer tomography, researchers took into account several additional variables: BMI, age, and sex. Thus, they developed a special mathematical formula: Score = 25 × CTR + 3 × sex + 1 × age/5 (years) + 0.2 × BMI. They proposed a CTR score ≥32 as the cut-off point, which allows for the diagnosis of cardiomegaly. To facilitate the calculations, an online calculator was offered (http://calc.chuv.ch/CTR (accessed on 8 May 2021)). The values calculated in this way are characterized by 84.04% sensitivity, 78.79% specificity, and 81.87% of correctly classified cases.

The researchers came to an interesting conclusion on the basis of earlier post-mortem CT research. They found large discrepancies in the CTR measurements on the CT pilot images and the measurements on the axial chest scans performed. Their conclusions indicated a very large upward overestimation of the CTR in the pilot CT scans; hence, these should not be trusted [29].

## 8. Conclusions

As demonstrated in the review above, the CTR as a simple, cheap, and quick tool still remains an important parameter in patient assessment in many clinical situations. In many cases, it has been shown that an increased CTR may be a good, initial parameter of the heart size preceding a more accurate assessment of the size of the heart and its cavities in echocardiography, computed tomography (increasingly available, but burdened with a much higher dose of ionizing radiation) and magnetic resonance imaging [30]. When aware of its limitations, it can be used in risk stratification, treatment assessment, or prognosis in various diseases, not only directly related to cardiology. Although it may seem that in 2021 talking about the clinical usefulness of the CTR may be an anachronism (mainly due to its low negative predictive value), according to the authors, given the discussed literature data, further research on the CTR is needed, especially regarding the comparison of the CTR with data from rapidly developing diagnostic methods such as multislice computed tomography and magnetic resonance imaging.
